# MR lung perfusion measurements in adolescents after congenital diaphragmatic hernia: correlation with spirometric lung function tests

**DOI:** 10.1007/s00330-021-08315-9

**Published:** 2021-11-06

**Authors:** Verena Groß, Katrin Zahn, Kristina Maurer, Lucas Wessel, Thomas Schaible, Stefan O. Schoenberg, Christel Weiß, Frank G. Zoellner, Meike Weis

**Affiliations:** 1 Department of Clinical Radiology and Nuclear Medicine, University Medical Center Mannheim, Heidelberg University, Theodor-Kutzer-Ufer 1-3, 68167 Mannheim, Germany; 2 Department of Pediatric Surgery, University Medical Center Mannheim, Heidelberg University, Theodor-Kutzer-Ufer 1-3, 68167 Mannheim, Germany; 3 Department of Neonatology, University Medical Center Mannheim, Heidelberg University, Theodor-Kutzer-Ufer 1-3, 68167 Mannheim, Germany; 4 Department of Medical Statistics and Biomathematics, University Medical Center Mannheim, Heidelberg University, Theodor-Kutzer-Ufer 1-3, 68167 Mannheim, Germany; 5 Computer Assisted Clinical Medicine, Mannheim Institut for Intelligent System, Medical Faculty Mannheim, Heidelberg University, Heidelberg University, Theodor-Kutzer-Ufer 1-3, 68167 Mannheim, Germany

**Keywords:** Perfusion imaging, Hernias, diaphragmatic, congenital, Magnetic resonance imaging, Lung, Spirometry

## Abstract

**Objectives:**

To evaluate whether lung perfusion continues to be reduced in 10-year-old children after congenital diaphragmatic hernia (CDH) and whether lung perfusion values correlate with spirometric lung function measurements.

**Methods:**

Fifty-four patients after CDH repair received dynamic contrast-enhanced (DCE) magnetic resonance imaging (MRI)-based lung perfusion measurements at the age of 10 years (10.2 ± 1.0 years). Additionally, a control group of 10 children has been examined according to the same protocol. Lung spirometry was additionally available in 43 patients of the CDH group. A comparison of ipsilateral and contralateral parameters was performed.

**Results:**

Pulmonary blood flow (PBF) was reduced on the ipsilateral side in CDH patients (60.4 ± 23.8 vs. 93.3 ± 16.09 mL/100 mL/min; *p* < 0.0001). In comparison to the control group, especially the ratio of ipsilateral to contralateral, PBF was reduced in CDH patients (0.669 ± 0.152 vs. 0.975 ± 0.091; *p* < 0.0001). There is a positive correlation between ipsilateral pulmonary blood flow, and spirometric forced 1-s volume (*r* = 0.45; *p* = 0.0024).

**Conclusions:**

Pulmonary blood flow impairment persists during childhood and correlates with spirometric measurements. Without the need for ionizing radiation, MRI measurements seem promising as follow-up parameters after CDH.

**Key Points:**

*• Ten-year-old children after congenital diaphragmatic hernia continue to show reduced perfusion of ipsilateral lung.*

*• Lung perfusion values correlate with lung function tests after congenital diaphragmatic hernia.*

## Introduction

Advances in understanding and treatment of congenital diaphragmatic hernia (CDH) have led to increased survival rates [1]. Nevertheless, this gain is associated with an increased degree of morbidity of survivors. This brings up the need for structured follow-up programs to recognize and potentially treat children after CDH. Focus on follow-up after CDH can also be observed in the increasing number of publications dealing with this topic.

Jesselstijn et al. published a survey of the CDH consortium members, which dealt with the question of how follow-up takes place in their hospital: There is a wide diversity concerning time-point and type of examination [2].

Despite differences in follow-up scheme, members of CDH consortium agree that follow-up needs to focus on the main problems children after CDH have, namely, feeding problems, developmental retardation in different areas of life, and lung function impairment. Additionally, the potential hernia recurrence has to be evaluated [2–4]. Most lung function measurements depend on the compliance of the child and are therefore limited in younger children. One image modality to measure lung function parameters is scintigraphy, which needs the application of ionizing radiation.

With magnetic resonance imaging (MRI), lung perfusion can be measured without ionizing radiation. Previous studies based on MRI perfusion measurements demonstrated for 2-year-old children that after CDH they show reduced values on the ipsilateral side. Additionally, 2-year-old children after ECMO requirement show even more reduced values [5–7]. It has not been evaluated whether ipsilateral perfusion defects persist during childhood and whether they correlate with lung function measurements.

Therefore, this study aimed to measure MR lung perfusion of adolescent children after CDH and correlate them with spirometric lung function measurements.

## Materials and methods

### Patients

Inclusion criteria for CDH patients for the study evaluation were (i) taking part in a local follow-up protocol), which includes MR lung perfusion measurements at the age of 10 to 12 years (10.2 ± 1.0 years); and (ii) sufficient image quality. Consequently, 54 patients (30 males, 24 females) could be included into this study. A total of 81.5% of patients suffered initially from left-sided and 18.5% from right-sided CDH. ECMO was required by 40.7% during the neonatal period.

Additionally, 10 controls (3 males, 7 females) without CDH have been included in this study (inflammation, *n* = 4; tumor, *n* = 3; neurological disorder, *n* = 2; trauma *n* = 1). Inclusion criteria for control group were (i) correct imaging protocol, (ii) no disease affecting lung perfusion, and (iii) correct patient age—which means an age interval of 10 to 12 years. The mean age of the control group was 11.4 ± 1.0 years. Perfusion imaging was included into the imaging protocol to visualize the cervical and thoracic vascular anatomy.

Local research ethics committee approved retrospective evaluation of study cohort and analysis of control group, respectively.

### MR imaging

All MR examinations have been performed on a single 3-T MRI system (Magnetom TimTrio, Siemens Healthineers). Lung perfusion imaging was included in the MR protocol, which consists of a head and thoracic examination. The entire examination took 25–30 min, 15 min of which for the thorax. A combination of coils (head, neck, and body phased-array) was used. No sedation was necessary.

A three-dimensional time-resolved angiography with stochastic trajectories (TWIST) sequence was applied for perfusion imaging [7]. TWIST view-sharing was set to 15% sampling density in the central and 20% in the outer region. Echo time was 0.78 ms and repetition time 2.28 ms with a flip angle of 14°. Generalized autocalibrating partially parallel acquisition (GRAPPA) of factor 3 was used. A temporal resolution of 1.5 s was chosen with an isotropic spatial resolution of 2 mm. Contrast agent (Dotarem, Guerbet) with a dosage of 0.05 mmol/kg body weight, diluted with the same volume of sodium chloride and followed by a sodium chloride bolus of 10 mL, was administered at a flow rate of 1 mL/s after the acquisition of five baseline images. Perfusion imaging was obtained under free breathing.

### Data analysis

Perfusion was quantified using a pixel-by-pixel deconvolution approach, which is implemented in an in-house–developed and certified OsiriX plugin [8, 9]. The arterial input function (AIF) was derived by a region of interest (ROI) placed in the main stem of the pulmonary artery. As quantitative pulmonary perfusion parameters, the pulmonary blood flow (PBF), the pulmonary blood volume (PBV), and the mean transit time (MTT) were calculated. The whole lung was manually delineated based on TWIST sequences and consequently a ROI that included whole lung has been calculated. Macrovessels of the hilar region have been excluded from the analysis. By applying this ROI on perfusion maps, mean values of PBF, PBV, and MTT per lung side could be calculated. Lung delineation was performed by two readers (1 year and 6 years experience in lung MRI) in agreement. The applied method has already demonstrated good inter- and intra-reader agreement in a previous study [5].

### Statistical analysis

All statistical calculations and graphical representations of data in this study were performed with a dedicated software (GraphPad Prism Version 8.3.0, GraphPad Software and SAS, SAS Institute Inc.). Lung perfusion parameters (PBF, PBV, MTT) of the ipsilateral versus contralateral side were compared and proven for significance by a paired *t*-test. Furthermore, differences in lung perfusion parameters were tested between the CDH group vs. control group, children with vs. without need for ECMO therapy, and children with vs. without clinically proven respiratory disorder. All of these comparisons were quantified and proven for significance by an unpaired *t*-test. The results of the spirometric lung function measurements were compared between children with the need for ECMO therapy and those without. This comparison was also quantified by an unpaired *t*-test.

Pearson’s correlation analysis was performed to detect a linear correlation between MRI-based lung perfusion parameters and spirometric measurements.

For all statistical tests mentioned above, a normal distribution was assumed, which was tested by the Shapiro–Wilk test.

In all tests, *p* < 0.05 was considered statistically significant.

### Lung function measurement

Spirometric measurements of CDH patients were performed on the same day as the MR examination. Due to retrospective approach of lung perfusion analysis, the control group did not receive lung function measurements. Results included the following values: forced expiratory volume per second (FEV1), forced vital capacity (FVC), Tiffeneau Index, which is the ratio of FEV1 and FVC, as well as the type of lung function impairment (restrictive, obstructive, combined, none). FEV1, FVC, and Tiffeneau Index were calculated in percentage of the nominal value and absolute terms.

## Results

### MR lung perfusion parameter

**Fig. 1:**
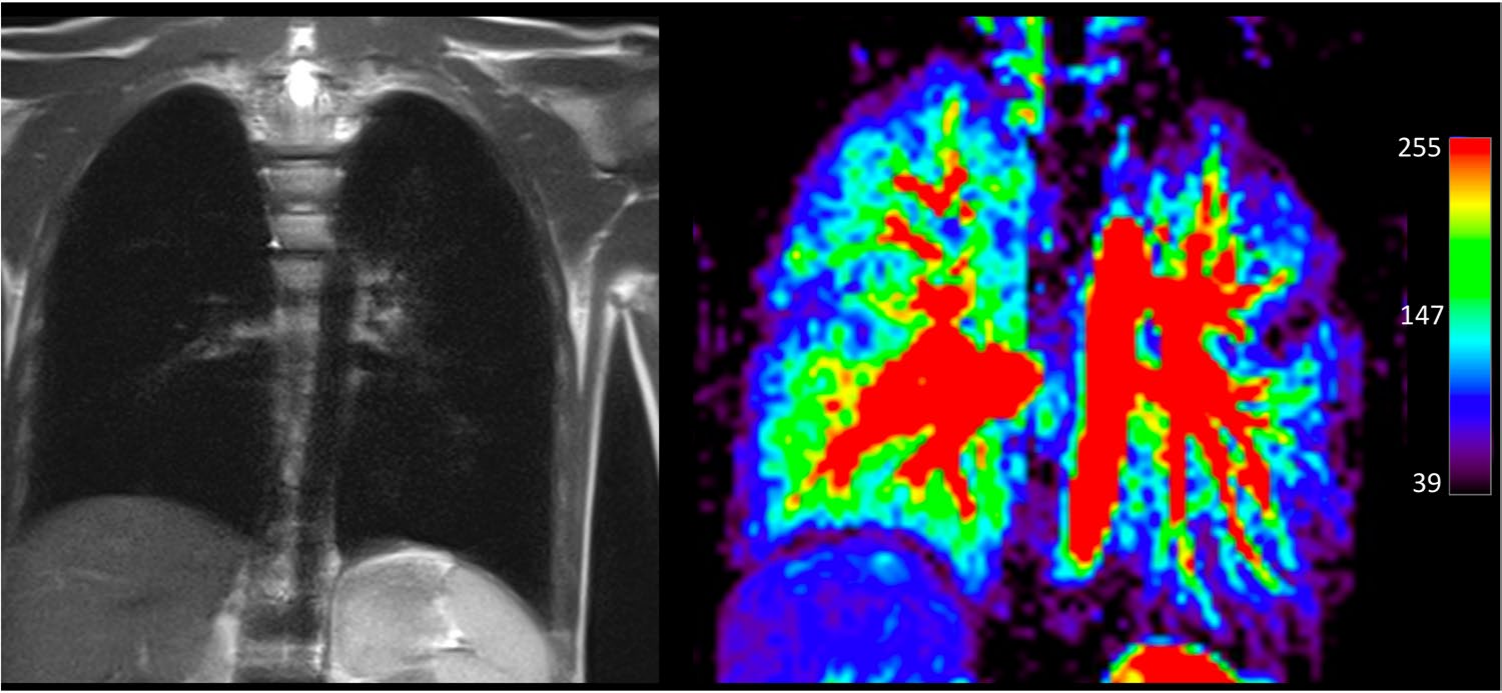
Ten-year-old patient after CDH repair on the left side. Left: Coronal T2-weighted image. Right: Pulmonary blood flow map. Already visually, reduced perfusion is recognized on the ipsilateral side. Color scale on the right codes perfusion values (mL/100 mL/min)

The reduction of the lung perfusion parameter can be observed already visually and qualitatively (Fig. 1).

Lung volume is significantly decreased on the ipsilateral side compared to the contralateral (*p* = 0.0012; Table 1). Lung perfusion parameters are significantly lower on the affected side than the contralateral, for example, mean ipsilateral PBF is 60.4 mL/100/mL/min compared to 90.0 mL/100/mL/min (*p* < 0.0001; Table 1).

**Table 1:** Comparison of lung volume and lung perfusion parameters between contralateral and ipsilateral lung in CDH patients

	Ipsilateral	Contralateral	*p* value
Lung volume (mL)	432.4 ± 128.6	481.0 ± 105.3	0.0012
Pulmonary blood flow (mL/100 mL/min)	60.4 ± 23.8	90.0 ± 26.5	< 0.0001
Pulmonary blood volume (mL/100 mL)	5.7 ± 2.2	8.1 ± 2.2	< 0.0001
Mean transit time (s)	6.3 ± 1.7	6.0 ± 1.3	0.0116

**Fig. 2:**
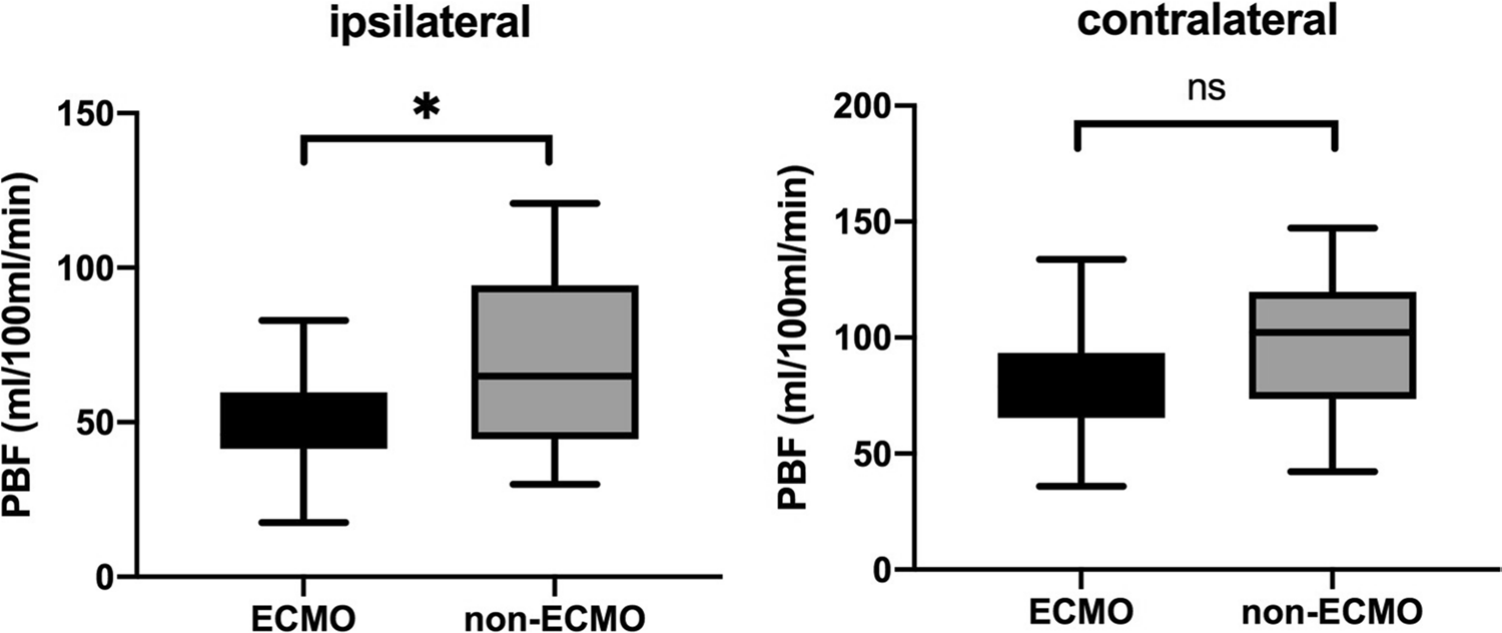
Comparison of pulmonary blood flow in 10-year-old CDH children with and without ECMO requirement

Ipsilateral pulmonary blood flow was significantly decreased in the ECMO group compared to children who did not require ECMO neonatally (49.7 ± 14.3 vs. 67.8 ± 26.1 mL/100 mL/min; *p* = 0.0056; Fig. 2). In the contralateral lungs, there is no significant difference in perfusion parameters between the ECMO and the non-ECMO groups (*p* = 0.2420).

**Fig. 3:**
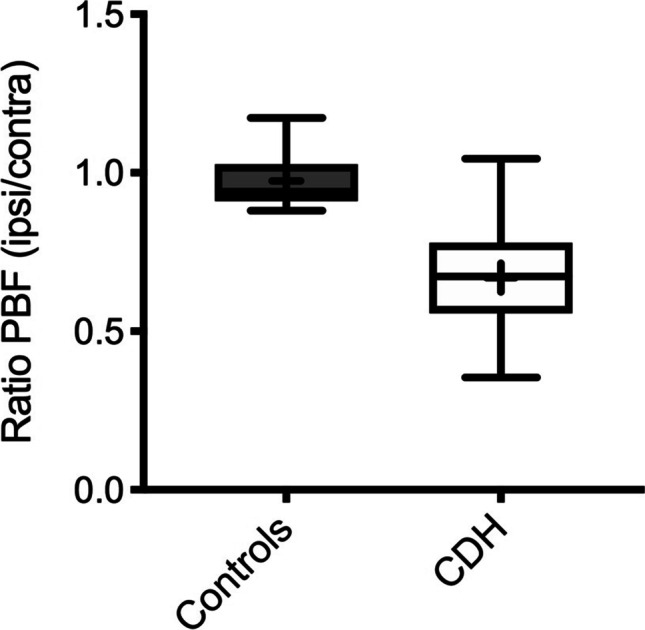
Comparative whiskers plot of pulmonary blood flow ratio (ipsilateral/contralateral) of CDH children and the control group

Pulmonary blood flow and pulmonary blood volume measurements were significantly lower on the ipsilateral side in children after congenital diaphragmatic hernia than those in normal controls (Table 2). The PBF ratio between ipsilateral and contralateral lung was close to 1 in the control group (0.975 ± 0.091) and accordingly lower in the CDH group (0.669 ± 0.152; *p* < 0.0001) (Fig. 3).

**Table 2:** Comparison of lung perfusion parameters between CDH patients and control group

	CDH	Controls*	*p* value
Lung volume ipsilateral	432.4 ± 128.6	462.3 ± 159.1	0.5207
Lung volume contralateral	481.0 ± 105.4	480.4 ± 160.2	0.9892
PBF ipsilateral (mL/100 mL/min)	60.4 ± 23.8	93.3 ± 16.09	0.0002*
PBF contralateral (mL/100 mL/min)	90.0 ± 26.5	95.4 ± 13.5	0.5326
PBF ratio (ipsi/contra)	0.669 ± 0.152	0.975 ± 0.091	< 0.0001*
PBV ipsilateral (mL/100 mL)	5.69 ± 2.19	7.92 ± 2.65	0.0128*
PBV contralateral (mL/100 mL)	8.08 ± 2.18	8.62 ± 2.00	0.4816
MTT ipsilateral (s)	6.33 ± 1.65	5.82 ± 0.95	0.5442
MTT contralateral (s)	5.95 ± 1.28	5.86 ± 0.77	0.7673

*CDH*, congenital diaphragmatic hernia; *PBF*, pulmonary blood flow; *PBV*, pulmonary blood volume; *MTT*, mean transit time

^*^Ipsilateral in control group means left, contralateral right lung

### Lung function

**Fig. 4:**
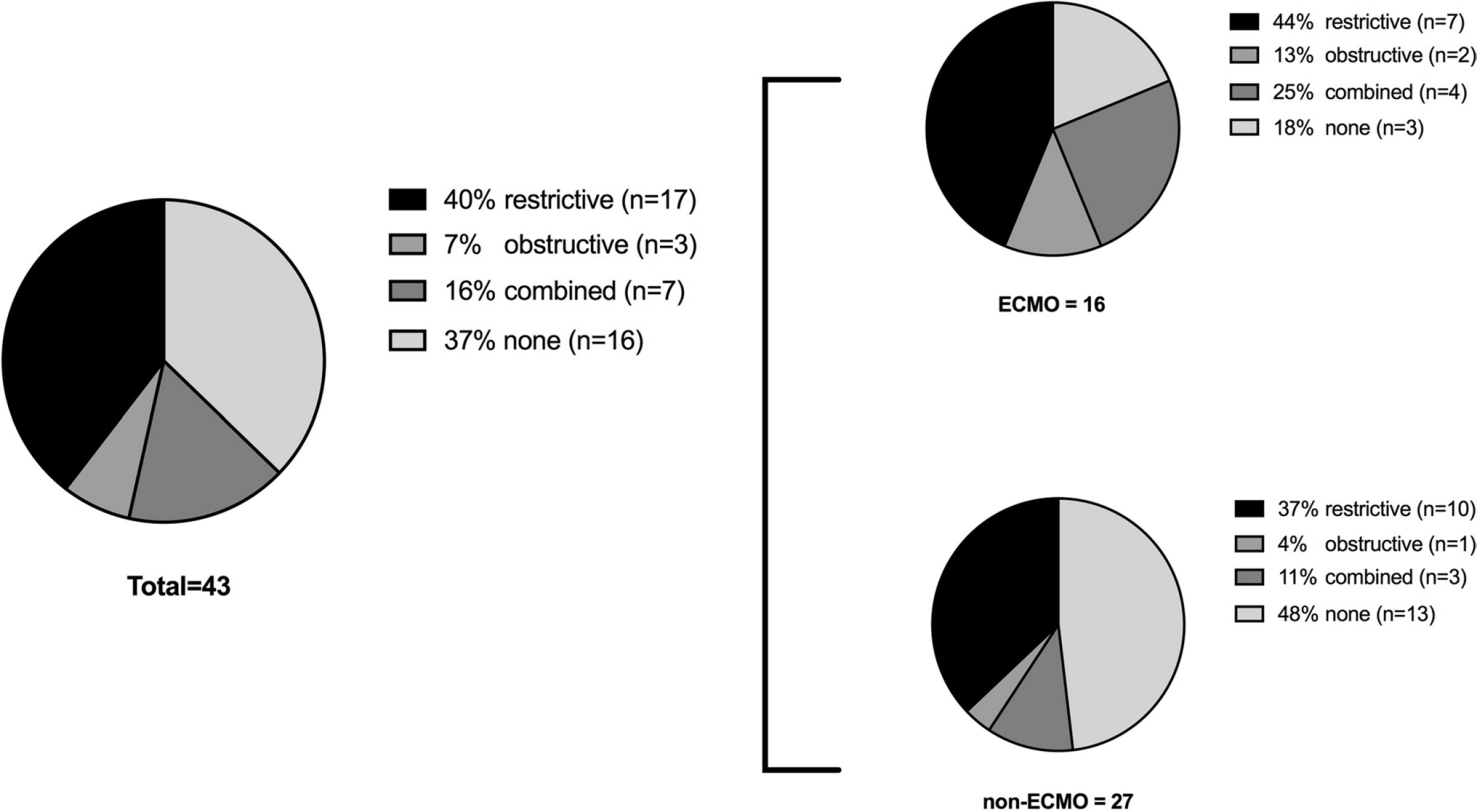
Distributions of respiratory disorders. On the left side, distribution of all children demonstrated. On the right side, distribution of ECMO and non-ECMO children is visualized

Lung function measurements were available in 43 children after CDH. Measurements showed that 17 (40%) patients suffered from restrictive respiratory disorder, 3 children (7%) from obstructive disorder, and 7 patients (16%) from a combination of both (Fig. 4). Sixteen (37%) patients showed normal lung function parameters. The mean forced expiratory volume for all children was 75.98 ± 22.82% of expected, and therefore lower than that in a healthy cohort. Forced vital capacity was also reduced with 74.79 ± 20.69% of expected.

A comparison of children with and without ECMO requirement showed that respiratory disorder was more frequently present in children with ECMO requirement (82%) than that in children without ECMO requirement (52%; Fig. 4).

### Correlation between MR lung perfusion parameter and lung function

**Fig. 5:**
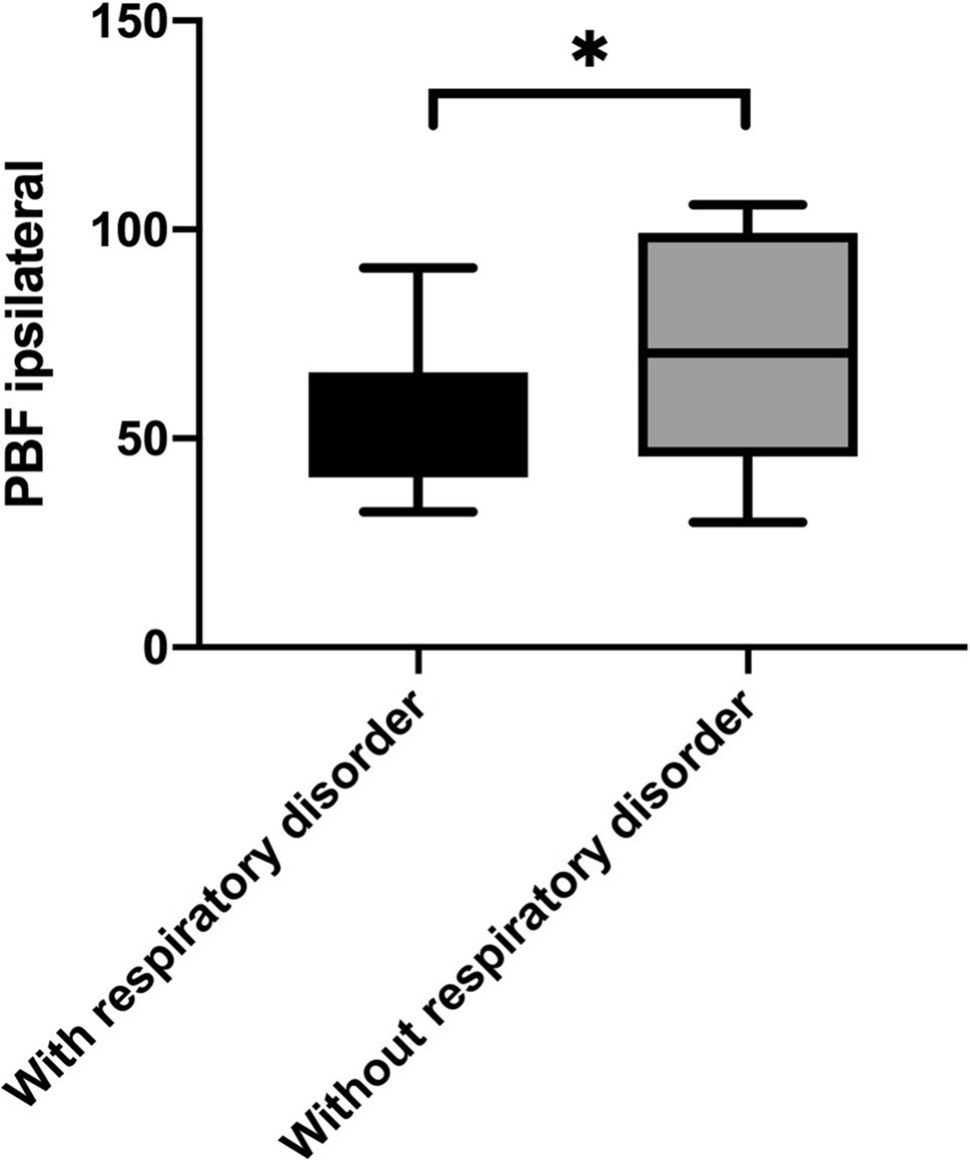
Difference of pulmonary blood flow between children with and without respiratory disorder

Ipsilateral PBF was significantly lower in children with respiratory disorder (53.8 ± 16.1 mL/100 mL/min) in comparison to that in children without respiratory disorder (70.9 ± 27.9 mL/100 mL/min; *p* = 0.0160; Fig. 5). Contralateral PBF showed no significant difference between children with and without respiratory disorder (85.4 ± 24.4 vs. 98.1 ± 27.9 mL/100 mL/min; *p* = 0.1272).

**Fig. 6:**
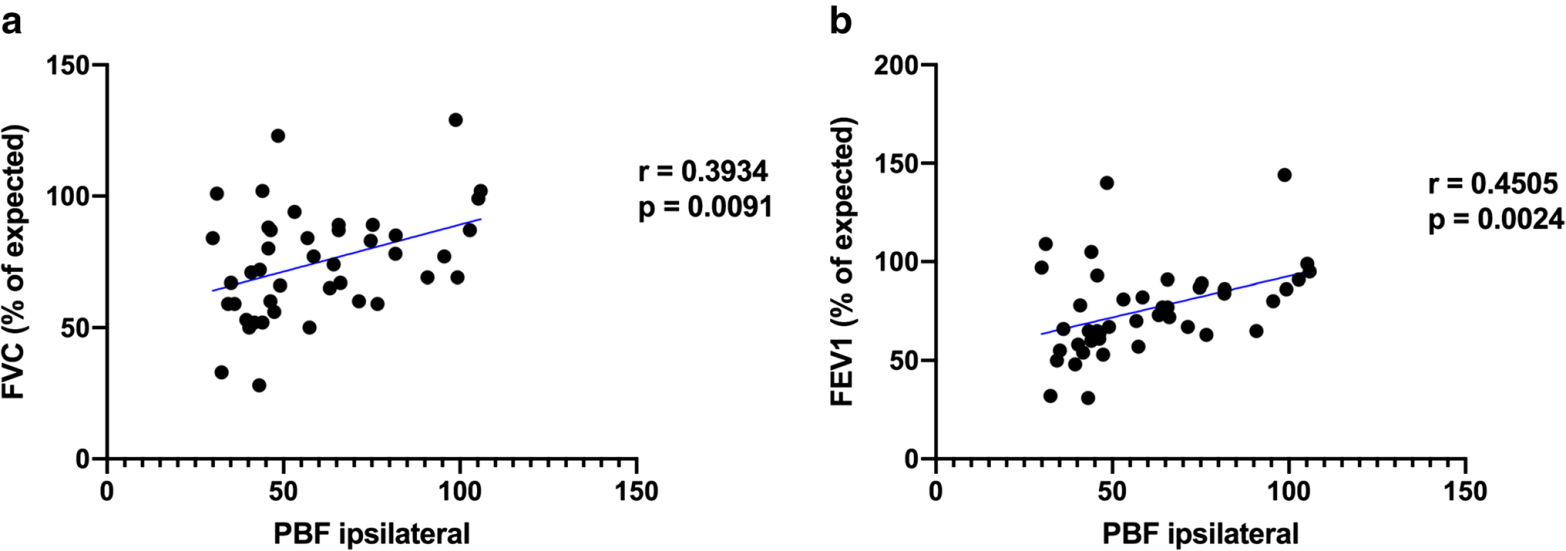
Correlation of pulmonary blood flow with spirometric results. **a** There is a positive correlation between ipsilateral pulmonary blood flow (PBF) and forced vital capacity (FVC). **b** There is as well a positive correlation between PBF and forced expiratory 1-s volume (FEV1)

There was a significant positive correlation between PBF ipsilateral and forced vital capacity (FVC; *r* = 0.39; *p* = 0.0091; Fig. 6) and forced expiratory volume (FEV1; *r* = 0.45; *p* = 0.0024, Fig. 6).

## Discussion

Adolescent children (10–12 years) after congenital diaphragmatic hernia (CDH) show reduced lung perfusion values ipsilateral, which can be detected by magnetic resonance imaging.

This difference in lung perfusion has been demonstrated previously in 2-year-old children after CDH [10]. In comparison to former studies, now older children have been examined. The present study demonstrates that ipsilateral perfusion deficits persist during childhood. According to present study, mean ipsilateral pulmonary blood flow (PBF) in 10-year-old children is 60 mL/100/mL/min and therefore similar to published values of 62 mL/100 mL/min in 2-year-olds [5]. The ratio of ipsi- and contralateral pulmonary blood flow is also comparable between 2-year-old children (0.69; [5]) and 10–12-year-old patients (0.67). Both the present and the previous study are based on cross-sectional data of 2-year- and 10–12-year-old children, respectively. Therefore, a direct comparison of absolute values is difficult. Future studies will need to address the individual course of lung perfusion longitudinally.

In accordance with other studies, the PBF ratio (ipsilateral/contralateral) of children after CDH differs significantly from the PBF ratio in healthy lungs, which is close to 1 [11, 12]. Therefore, the PBF ratio of ipsilateral to contralateral lung could serve as a parameter to grade the degree of lung perfusion impairment.

As demonstrated for 2-year-old children [6], children who required extracorporeal membrane oxygenation (ECMO) therapy in their neonatal period show even more reduced lung perfusion values on the ipsilateral side. ECMO therapy is only performed in case of respiratory or hemodynamic failure associated with severe pulmonary hypertension; and therefore, status after ECMO is a sign of CDH severity. The current study demonstrates that structural changes in vascular bed—represented by lung perfusion—are not regenerated during childhood but persist until the age of adolescence. This result is in accordance with literature demonstrating that children after ECMO therapy suffer from higher morbidity [13–16].

Scintigraphical measurements have also depicted perfusion deficits. In scintigraphy, lower ventilation to perfusion coefficients are present. Bjorkman et al. showed that 6-month-old infants have a variable degree of ventilation-perfusion abnormalities, which are correlated with the persistence of pulmonary hypertension [17]. Stefanutti et al. investigated several outcome parameters in 8-year-old CDH patients. Among others, they found reduced perfusion values on the ipsilateral side [18]. Hayward et al. investigated changes in ventilation/perfusion (V/Q) mismatch during childhood in serial scintigraphical measurements: They could demonstrate that some survivors develop severe V/Q mismatch during childhood [19]—these children are at risk for severe pulmonary morbidity and need intensive follow-up. In our study, we could also demonstrate that perfusion deficits, which could be depicted in 2-year-old children, persist until the age of 10. However, intraindividual changes have not been evaluated yet. Pal et al. evaluated serial perfusion studies at the ages of 3 months, 9 months, and 6 years [20]. Thereby, they found that lung perfusion is reduced in the ipsilateral lung but increases during childhood, associated with amelioration of lung function parameters. In contrast to scintigraphical methods, MR perfusion measurements are feasible without ionizing radiation and seem promising as a follow-up tool. Future studies are needed to evaluate the individual course of lung perfusion, measured by MRI. A promising development in this context is the implementation of perfusion measurements without the need for a contrast agent. One technique, which has already been used to study pulmonary perfusion, is the Fourier decomposition technique [21, 22]. Recently, a further development of this technique, has been used in patients after cystic fibrosis [23, 24]. To our knowledge, a transfer of this technique to younger patients with associated technical challenges has not been performed yet but seems promising.

Lung perfusion measurements have also been performed intrauterine by sonographic measurements. Thereby, it could be demonstrated that children with CDH show reduced lung perfusion values associated with increased pulmonary artery resistance already prenatally [25]. In combination with the present study, these results show that a hypoplastic or dysfunctional vascular bed is one key in the further understanding of morbidity in congenital diaphragmatic hernia.

Another—or a related—part of morbidity after CDH is the presence of lung function morbidity. The current study demonstrates that adolescent children after CDH suffer in around 40% from restrictive, in 16% from combined, and in 7% from obstructive lung function impairment. A total of 37% have average spirometric results. Both forced expiratory volume (FEV) and forced vital capacity (FVC) were reduced in the study cohort. These results are in good agreement with a study of Majaesic et al., which also demonstrates reduced FEV and FVC values in 8-year-old children after CDH [26].

Similarly, a study that investigated 13-year-old children also showed (besides others) reduced values of FEV [27, 28]. Interestingly, an investigation of 24-year-old CDH survivors could demonstrate reduced FEV values in some survivors, but this was not associated with reduced quality of life [29]. This is in agreement with the results of Arena et al.—despite persistent measurable FEV deficits, symptoms of obstruction disappeared [30]. These results raise, of course, the question of whether persistent airway flow impairment measurements are accompanied by respiratory problems later in life, for example, in their forties. This question cannot be answered yet. During early childhood, CDH survivors suffer from recurrent respiratory tract infections and have a higher risk of developing asthma [28, 31]. In our study cohort, significant differences between CDH patients with neonatal ECMO therapy as compared to those without ECMO therapy could be detected concerning severity of ipsilateral perfusion deficits and respiratory disorders.

The current study shows a relationship between ipsilateral pulmonary blood flow and both expected FEV1 and expected FVC. Therefore, MR perfusion values correlate with respiratory measurements. On the one hand, this can be explained by structural lung changes of vascular bed and airways that persist during childhood [32]. On the other hand, lung ventilation and perfusion are associated with several mechanisms, of which the Euler-Liljestrand is the most popular [33].

One limitation of this study is the restricted number of patients and normal controls, which is mainly owed by retrospective study design. Another limitation of this study is the manual placement of arterial input function and manual lung delineation—future studies should help transfer automatic lung segmentation, which have been proposed by several authors [34, 35] to clinical routine. Another limitation of the present study is that the comparison of mean perfusion values per lung side eliminates the possibility of comparing regional differences or distribution patterns. Future studies should address this question potentially by applying histogram-based analyses.

To summarize, MR lung perfusion deficits persist at least until the age of 10 years after CDH. MR perfusion measurements correlate to lung function measurements without the need for compliance during measurement. Therefore, MR perfusion measurement could be a tool for stratifying the need for more intensive or longer follow-up in children after CDH without ionizing radiation. This would be of advantage especially for high-risk patients with CDH requiring neonatal ECMO therapy.

## Abbreviations

CDH: Congenital diaphragmatic hernia
ECMO: Extracorporeal membrane oxygenation therapy
FEV1: Forced expiratory volume per second
FVC: Forced vital capacity
GRAPPA: Generalized autocalibrating partially parallel acquisition
MRI: Magnetic resonance imaging
MTT: Mean transit time
PBF: Pulmonary blood flow
PBV: Pulmonary blood volume
TWIST: Time-resolved angiography with stochastic trajectories

## Declarations

### Guarantor

The scientific guarantor of this publication is PD Dr.med. Meike Weis, MSc.

### Statistics and biometry

One of the authors has significant statistical expertise (Prof. Dr. Christel Weiß).

### Study subjects or cohorts overlap

Fifteen patients of our current cohort (54 patients) have been examined at the age of 2 years. Four of them with identical study protocol. Now, they have been re-examined at the age of 10 years.

### Methodology

• retrospective

• diagnostic or prognostic study

• performed at one institution
